# The growing need for controlled data access models in clinical proteomics and metabolomics

**DOI:** 10.1038/s41467-021-26110-4

**Published:** 2021-10-01

**Authors:** Thomas M. Keane, Claire O’Donovan, Juan Antonio Vizcaíno

**Affiliations:** European Molecular Biology Laboratory, European Bioinformatics Institute (EMBL-EBI), Wellcome Trust Genome Campus, Hinxton, Cambridge CB10 1SD UK

**Keywords:** Proteomics, Data publication and archiving, Medical genomics, Metabolomics, Molecular medicine

## Abstract

More and more clinical studies include potentially sensitive human proteomics or metabolomics datasets, but bioinformatics resources for managing the access to these data are not yet available. This commentary discusses current best practices and future perspectives for the responsible handling of clinical proteomics and metabolomics data.

## Current data sharing practices in proteomics and metabolomics

Proteomics and metabolomics approaches are increasingly used in clinical research. This trend is fueled by rapid technical developments in both fields and the growing importance of clinical multi-omics studies, which combine proteomics, metabolomics and/or different types of DNA/RNA sequencing methods to characterize complex phenotypes.

The main analytical approach used in both proteomics and metabolomics is mass spectrometry (MS). Other approaches include established methods such as NMR-based metabolomics and antibody-based protein assays (e.g. protein arrays) as well as emerging techniques, e.g. the use of affinity reagents for proteomics (e.g. Somalogic^®^ and Olink^®^ assays).

In parallel to the many technical developments, open data policies in proteomics and metabolomics have greatly advanced over the last few years, following the trend established by genomics and transcriptomics. It is now commonplace to store proteomics data that support the conclusions in scientific publications in public data repositories. In 2011, the most prominent proteomics resources came together to collaborate formally within the ProteomeXchange (PX) consortium^1^ (http://www.proteomexchange.org/), resulting in standardized data submission and dissemination practices. At present, there are six PX members: the PRIDE database in Europe, PeptideAtlas/PASSEL, MassIVE and Panorama Public in the USA, jPOST in Japan and iProX in China. Of those, PRIDE is the most used resource, storing more than 80% of all PX datasets.

In metabolomics, the trend towards open data sharing has been slower due to a number of factors, predominantly that spectral libraries, critical for metabolite identification, remain expensive to generate. Therefore, in-house spectral libraries are still kept private by many research laboratories and nearly all commercial metabolomics service providers. Some commercial providers, who are frequently recruited by clinicians to perform metabolomics experiments as part of clinical studies, tend to protect their intellectual property by sharing neither the spectral libraries or the MS raw data. Still, metabolomics data submissions have increased exponentially over the last three years, a development driven by the community, funders and journals. This has led to a rapid growth of the pre-existing data resources MetaboLights (Europe) and Metabolomics Workbench (USA), both established in 2012, and the creation of MetaboBank (Japan) in 2021. All three resources share their data through MetabolomeXchange (http://www.metabolomexchange.org) which has facilitated greater dissemination. MetaboLights has also established collaborations with metabolomics service companies such as Metabolon in order to make more data available. The development of common submission practices in metabolomics, however, is a work in progress.

## Ethical considerations for proteomics and metabolomics data

The proteomics and metabolomics resources mentioned above support fully open data sharing. However, this model is incompatible with the requirements for sharing patient identifiable information (also known as ‘personal data’) collected through healthcare or research projects. Such personal data (including genetic information) hold the potential to re-identify research participants. Therefore, these data are typically only shared with authorized researchers, in compliance with participant consent data use conditions and/or applicable personal data regulations to protect citizen privacy, e.g. the European Union General Data Protection Regulation (GDPR) or national laws governing the sharing of healthcare data. Currently, these (usually genetic) data are mainly made available through resources that support the storage and dissemination of human controlled access data such as the European Genotype-phenome Archive (EGA), the database of Genotypes and Phenotypes (dbGAP) in the USA and the Japanese Genotype-phenotype Archive (JGA). Here, potential data users apply for access via the relevant Data Access Committee (DAC). The DAC confirms the identity and research credentials of the applicant, and that the proposed research is allowable under the data use conditions of the dataset.

The necessity of similar controlled access options for proteomics and metabolomics data will depend on whether these data can potentially be used to identify research participants—a question that has already been thoroughly explored for genetic data^2,3^, which are thus considered to be patient identifiable information by data protection regulations^4^. Furthermore, controlled access to proteomics and metabolomics data may become necessary because of requirements related to patient consent, personal data regulations like GDPR or any other relevant legislation.

High-throughput proteomics platforms have rapidly evolved in recent years and now have the potential to collect sufficient data to make single individual personally identifiable^5^. Currently, potentially sensitive proteomics datasets of clinical interest are routinely made openly available via the PX resources, but ethical discussions over clinical proteomics data have begun recently^5,6^. At PRIDE, we are receiving an increasing number of queries about data management of sensitive human data. In fact, we had to withdraw a small number of non-released (private) datasets because of the advice provided to the data submitters by their data protection officers. The number of sensitive human datasets that, due to these requirements, have not been submitted is difficult to estimate. However, more valuable datasets could be lost to the community unless an appropriate controlled-access infrastructure is implemented.

A key difference between genomic and proteomic data is the lower sequence coverage in proteomics, resulting in a much lower number of observed variants and reduced identifiability. The highest risk to render individuals identifiable by proteomics lies in the MS raw data and the amino acid sequence database. In addition, re-identification of individuals was recently reported based on protein expression information from plasma samples^5^, even without variant sequence information at the protein level. In a recent community-driven paper^6^, the potential of different proteomics data types to provide patient identifiable information was discussed. This could trigger access restrictions tailored for different proteomics data types in the future.

Metabolomics assays are firmly established in human medicine, e.g. in the form of glucose measurements for diabetes or newborn blood spot screening for genetic disorders. Its utility for predicting the development of disease, monitoring treatment efficacy, identifying disease-causing metabolites and even suggesting dietary changes to aid therapy^7^ has been recognised more recently. As a result, metabolomics has begun to move out of purely fundamental research into the wider healthcare environment, accelerating the discussions over data sharing and the related potential ethical issues. Concerns related to identifiability also apply to metabolomics data. For instance, a metabolic disorder theoretically can suggest rare diseases or unusual medication, which might be sufficient to identify an individual. Potentially sensitive metabolomics datasets are, at present, either being retained at source (and not available to anyone except the original researchers and healthcare providers) or partially deposited in open resources such as MetaboLights.

Further research is needed to obtain more definitive scientific evidence for the identifiability potential of the main data types and analysis workflows in proteomics and metabolomics. The conclusions should then be used as to inform future policy decisions and develop tailored guidelines for sensitive proteomics and metabolomics datasets, leveraging the existing ones for DNA/RNA sequencing data.

## Paths toward tailored bioinformatics infrastructures

A few proteomic and metabolomic datasets are already available under controlled access. Examples include proteomics data deposited in the Alzheimer’s disease (AD) knowledge Portal (https://adknowledgeportal.synapse.org/)^8^, antibody-based and MS-based metabolomics studies made available via national trusted research environments (e.g. Genomics England) as well as imaging and proteomics datasets deposited in the EGA as part of multi-omics studies (e.g. EGAS00001004349, EGAS00001003179). However, existing controlled access resources such as EGA, dbGaP and JGA are not ideal for proteomics and metabolomics datasets. The data model of these resources is based on the Sequence Read Archive data model, which is tailored for sequencing-based assays and cannot appropriately represent proteomics and metabolomics datasets, limiting interoperability and findability.

For the reasons explained above, there is a growing demand from scientists, funders and scientific journals to have a controlled access infrastructure for sensitive human non-DNA omics data. In our view, addressing this need will require implementation of approaches that are tailored to different geographical areas. For Europe, we envision that submission guidelines and data models for human sensitive datasets will be based on those available for proteomics and metabolomics open datasets. Therefore, proteomics and metabolomics controlled access datasets will be both accessible and linked via EGA and the field-specific resources (PRIDE and MetaboLights), which already provide domain-specific data models and systems for the submission, access and visualisation of MS data and have strong relationships with their respective communities. In the USA, it is envisaged that open metabolomics data will be made available in the National Metabolomics Data Repository, which will be mandated by NIH data sharing policies as of 2023. Personal identifiable data will be linked but will only be accessible to approved researchers in a separate controlled access environment. In Japan, it is not expected that MetaboBank will develop a controlled access version. However, there have been discussions about the development of a specific repository for personal data including genomic and other omics information for medical use (not for research). In China, the “Regulations of the People’s Republic of China on the Management of Human Genetic Resources” were implemented on July 1st, 2019. Since the formal announcement of the regulations, the Beijing Proteomics Research Center’s Genetic Information Preservation Database (dbPDPM) and the Chinese National Population Health Data Center have been authorised to collect, preserve, utilize and provide the Chinese human genetics resource. It is planned that dbPDPM, which will be an extension of iProx to support multi-omics data, will be launched at the end of 2021 or early 2022. At the time of writing, there are no immediate plans to develop controlled access versions of the local PX resources in the USA (PeptideAtlas/PASSEL, MassIVE, Panorama Public) or Japan (jPOST), but this may change depending on the availability of funding.

Complementarily, we argue for an approach rooted in international collaboration and building on existing efforts for coordinating standardized submission and access to open datasets. At the level of each specific field (PX and MetabolomeXchange resources), it would be desirable to extend the existing open data models toward mappings into a clinical schema and ontologies, which would enable data discovery across multiple repositories for a given clinical profile. This would be fully compliant with the FAIR (Findable, Accessible, Interoperable, Re-usable) principles as well as individual countries’ policies. Going even further, a common data model for human sensitive omics datasets (including DNA/RNA sequencing, proteomics, metabolomics, and imaging) would enable queries across different domains and the linking of multi-omics studies.

In addition to MS data, non-MS proteomics techniques based on the use of affinity reagents are becoming more popular. Since these approaches are not yet generalised and the current PX data model only supports MS experiments, tailored repositories for these data types are lacking. However, studies using these approaches may also be subject to controlled access restrictions (e.g. in case of^9^). Future extensions of the existing PX resources, together with guidelines for metadata and dedicated software tools, will have to be developed to support these non-MS experiments.

## Decentralisation as a challenge for clinical data sharing

It is important to highlight that the traditional field-centric archives are not the only relevant parties in these developments. National repositories for specific geographical areas (e.g. countries and/or regions within those countries) will be an increasingly important source of human cohort data for research. Large human cohort data generated by researchers and national or regional healthcare initiatives are emerging. As a consequence, most developed countries now have nascent personalised medicine programmes^10^. Thus, human multi-omics is undergoing a step change from being a predominantly research-driven activity to being funded through healthcare systems. Data generated in a healthcare context will be subject to more stringent information governance than research data. This is already becoming apparent for genetic data; for example, Genomics England requires all research to be carried out within their trusted research environment with strict controls on export of research results.

The decentralisation of human omics data for research presents significant risks of human research data becoming siloed and undiscoverable. Efforts such as the Beacon Network^11^ and the IHCC (International HundredK+ Cohorts Consortium) Cohort Atlas aim to address the cohort discovery and access challenge. The EGA is transitioning from a centralised model to become a federated network of nodes where individual level data can remain within the regional or national borders to comply with local regulations. The Federated EGA aims to implement the FAIR principles across an international network of human data repositories and analysis platforms to enable discovery and harmonised data access, built upon international standards such as those from the GA4GH (Global Alliance for Genomics and Health).

## Outlook

There is a growing need for bioinformatics infrastructure and policy in metabolomics and proteomics to address the requirements of patient identifiable human data. This is a logical consequence of the maturation and wider use of these experimental approaches in biomedicine. We encourage that data privacy experts, interested researchers and data resource managers in both fields get together to share their knowledge and develop policy. Additionally, if feasible, they should define objective metrics that can inform researchers, legislators and the society as a whole of the privacy risks associated with the different proteomics and metabolomics data types.
